# Changes in Resting-State Functional Connectivity of the Hippocampus Following Cognitive Effort Predict Memory Decline at Older Age—A Longitudinal fMRI Study

**DOI:** 10.3389/fnagi.2019.00163

**Published:** 2019-07-16

**Authors:** Noga Oren, Elissa L. Ash, Irit Shapira-Lichter, Odelia Elkana, Osnat Reichman-Eisikovits, Lior Chomsky, Yulia Lerner

**Affiliations:** ^1^Functional MRI Center, Beilinson Hospital, Rabin Medical Center, Petach Tikva, Israel; ^2^Sackler Faculty of Medicine, Tel Aviv University, Tel Aviv, Israel; ^3^Department of Neurology, Tel Aviv Sourasky Medical Center, Tel Aviv, Israel; ^4^Behavioral Sciences, Academic College of Tel Aviv-Yaffo, Tel Aviv, Israel; ^5^Sagol Brain Institute Tel Aviv, Tel Aviv Sourasky Medical Center, Tel Aviv, Israel; ^6^Sagol School of Neuroscience, Tel Aviv University, Tel Aviv, Israel

**Keywords:** aging, episodic memory, functional magnetic resonance imaging, memory deficit, follow-up

## Abstract

Memory decline is a feature of some, but not all, healthy older adults. The neural patterns of this variability are still largely unknown. We examined the resting-state functional connectivity (RSFC) of older and younger adults before and after cognitive effort as an underlying feature for subsequent memory changes, focusing on the RSFC between the left anterior hippocampus (laHC) and the posterior hippocampi (pHC). Results showed that for younger adults, post-effort increases in laHC–pHC RSFC were related to increases in RSFC between the laHC and the hubs of the default mode network (DMN). However, for older adults, post-effort increases in the RSFC of laHC–pHC were related to decreases in the RSFC of the laHC and right precentral gyrus. Thus, the correlation between intra-HC and inter-HC RSFC was altered with cognitive effort and aging. Importantly, older adults who had lower post-effort RSFC between the laHC and the pHC demonstrated a decline in episodic memory 2 years later. Hence, the change in intra-HC RSFC following cognitive effort was able to predict subsequent memory function with aging in our sample.

## Introduction

Older adults are especially impaired in episodic memory (Grady, 2012). Extensive studies have delineated the phenomenon of age-related memory decline (Nyberg et al., 2012), as well as the structural and functional brain changes (Grady, 2012) and the trajectory of accumulating neuropathologies with age (Ewers et al., 2011). Yet, researchers and clinicians are still unable to predict future decline in memory function in cognitively intact older adults. This prediction is crucial to maintain quality of life with advancing age, since it can potentially allow for a timely initiation of interventions. To investigate the issue, we used functional magnetic resonance imaging (fMRI) and concentrated on two features that were selected based on previous studies. First, we focused on the hippocampus (HC) and its functional networks, as they are known to be related to changes in episodic memory with age (Andrews-Hanna et al., 2010, 2014; Moscovitch et al., 2016). Second, we examined resting-state functional connectivity (RSFC) patterns, since they allow for detection of even minor changes that affect the flow of information between regions and networks (Ferreira and Busatto, 2013). Specifically, we examined changes in RSFC before and after cognitive effort, since cognitive effort can potentially exhaust compensatory mechanisms that conceal hidden deficits (Steffener and Stern, 2012). Thus, the difference between RSFC before and after cognitive effort is anticipated to have sensitivity to predict future decline in the integrity of neural networks with aging. We will elaborate on these two features below.

The HC is a key brain structure in memory (Moscovitch et al., 2016) that undergoes pronounced changes with aging (Fjell et al., 2014). The anterior and posterior sections of the HC have distinct structural and functional characteristics, which are affected differently by aging (Poppenk et al., 2013; Strange et al., 2014; Moscovitch et al., 2016) and contribute to distinct aspects of memory. The anterior HC (aHC) is more related to encoding, response to novelty, pattern completion, and capturing global aspects of an event (Poppenk et al., 2013; Strange et al., 2014; Moscovitch et al., 2016). The posterior HC (pHC) is more related to retrieval, response to repeated presentation of a stimulus, pattern separation, and capturing detailed information. Many studies have examined the differences in RSFC of the aHC and pHC with other regions (Wagner et al., 2016) and how RSFC changes with age (Salami et al., 2016). In the current study, we were interested in the RSFC between the aHC and the pHC (to be termed intra-HC RSFC along the long HC axis), which is reported to be reduced at older age (Damoiseaux et al., 2016), yet the functional manifestation of this reduction is still unclear.

The HC operates in concert with the default mode network (DMN; Buckner et al., 2008), yet these connections decrease at older age (Ferreira and Busatto, 2013; Salami et al., 2014). The DMN is composed of a core and two distinct subsystems: the medial temporal lobe (MTL) and the dorsomedial prefrontal (dmPFC) subsystems (Andrews-Hanna et al., 2010). The core includes two cortical midline regions, namely, the posterior cingulate cortex (PCC) along with the precuneus, and the ventromedial prefrontal cortex (vmPFC). These regions exhibit strong functional connectivity (FC) with the DMN subsystems and serve as hubs across the brain (Buckner et al., 2009). The MTL subsystem is composed of the HC, ventral medial prefrontal cortex (a section below the anterior cingulate cortex), inferior parietal lobule (IPL), retrosplenial cortex, and parahippocampal cortex (Andrews-Hanna et al., 2010). The MTL subsystem is essential for memory (Andrews-Hanna et al., 2010, 2014; Moscovitch et al., 2016).

RSFC patterns are understood to constitute traces of task-evoked coactivation among regions (Kelly and Castellanos, 2014), along with inherited personal tendencies, meaning the structural and functional biases of an individual’s nervous system, both innate and developed across the life span (Harmelech and Malach, 2013). As such, RSFC may provide insights about an individual’s traits and neuropathologies. With advancing age, the DMN (Andrews-Hanna et al., 2007) and the HC (Toussaint et al., 2014) are usually reported to have lower RSFC, which is not explained by a decrease in gray matter volume (Ferreira and Busatto, 2013). Lower RSFC between the HC and the DMN hubs is associated with lower memory performance at older age (Wang et al., 2010; He et al., 2012). With regard to RSFC and older age, we wish to emphasize two findings. First, older adults exhibit greater RSFC between the left and right HC, which is associated with lower RSFC between the HC and the DMN (Salami et al., 2014). The second finding is that RSFC is altered by cognitive effort (Grigg and Grady, 2010), a feature that can be harnessed to better differentiate between age groups. Specifically, Jacobs et al. (2015) found that younger adults show higher RSFC patterns in the DMN following encoding, while older adults do not. Here, we tried to combine the findings of Salami et al. (2014) and Jacobs et al. (2015) to examine whether cognitive effort affects the association between intra-HC RSFC and RSFC of the HC with the hubs of the DMN, and if that association is altered with age. Moreover, based on findings that RSFC of the HC predicts memory function in older adults (Wang et al., 2010; He et al., 2012), we also examined whether changes in intra-HC RSFC following effort could predict future memory decline with age.

The study had three aims. The first aim was to characterize the effects of age and cognitive effort on RSFC of the HC. To this end, older and younger adults completed an fMRI scan, which included two resting-state runs separated by a long cognitive task (composed of cycles of encoding, mathematical distraction, and recognition). Note that we refer to the period of the cognitive task as “cognitive effort” since it includes both memory and nonmemory tasks. When a particular task from that period is analyzed, the task itself is stated specifically in the text. A seed-based RSFC analysis was used, with the seed in the HC. The second aim was to examine whether different HC connections changed following cognitive effort in similar ways. This aim was achieved by evaluating the correlations of the differences in RSFC before and after effort for distinct HC connections. We also examined whether these associations changed with age. Specifically, we focused on the association between RSFC of the aHC and pHC and between the RSFC of the HC and the hubs of the DMN. The third and final aim was to examine whether changes in intra-HC RSFC following effort may predict future memory decline in older adults. To fulfill this aim, the older adults completed three neuropsychological evaluations, one at the time of the fMRI scan (baseline) and each subsequent year for 2 years (follow-ups), and their performance was correlated with the changes in intra-HC RSFC following effort identified at baseline.

## Materials and Methods

### Participants

Twenty-five younger [age range: 21–35 years; mean age standard deviation (SD)]: 29 years (4); mean education: 15.24 years (2.29); nine females and 28 older (age range: 65–79 years; mean age: 71.8 years (4.6); mean education: 17.1 years (3.05); 16 females) healthy adults participated in the study. Gender was excluded as a potential confounding factor (see Supplementary Results). All participants were right-handed, except for two younger participants who were ambidextrous with a tendency to use the right hand more, as indicated by the Hebrew translation of the Edinburgh Handedness Inventory (Oldfield, 1971). All participants were native or fluent Hebrew speakers. None had a history of neurological or psychiatric problems, and all reported normal or corrected to normal vision. None of the older adults reported substantial cognitive decline during a screening interview; all had intact cognitive function, as indicated by a comprehensive neuropsychological assessment (see details below); and none was diagnosed with mild cognitive impairment or dementia using established criteria (Petersen, 2004; American Psychiatric Association, 2013). The experimental procedures were approved by the Tel Aviv Sourasky Medical Center Institutional Review Board, and all participants provided written informed consent.

### Neuropsychological Assessment at Baseline and Follow-Up

Older adults completed a comprehensive neuropsychological assessment on three occasions. The first was the baseline assessment, conducted during a separate meeting shortly before the fMRI scan. The second was conducted approximately 1 year after the baseline assessment (to be termed “1y follow-up”), and the third was conducted approximately 2 years following the baseline assessment (to be termed “2y follow-up”). The baseline sample included 28 participants; one participant was excluded at the 1y follow-up due to failure to make contact. In each assessment, the following tests were administered (all in Hebrew): Montreal Cognitive Assessment (MoCA; Nasreddine et al., 2005), Rey Auditory Verbal Learning Test (RAVLT; Vakil and Blachstein, 1997), logical memory from the Wechsler Memory Scale (WMS), Rey–Osterrieth complex figure (Lezak et al., 2004), phonemic and semantic verbal fluency tests (Kavé and Knafo-Noam, 2015), digit span and general knowledge tests from the Wechsler Adult Intelligence Scale (WAIS), and Trail Making Test (TMT) parts A and B (Lezak et al., 2004). All tests except the MoCA were scored using norms for age. Raw scores were used for the MoCA test since there are no formal norms for age (Oren et al., 2015). The assessment confirmed the intact cognitive status of the participants at all time points (i.e., no more than one test with a score of 1.5 SD below age-matched norms; Table 1). Younger participants completed only the RAVLT, verbal fluency, and digit span after the fMRI scan (Table 1). Details regarding a subset of the older and younger adults can also be found in Oren et al. (2015), Elkana et al. (2016) and Oren et al. (2017a).

**Table 1:** Results of neuropsychological assessment of older and younger adults.

	Older group, baseline	Older group, 1-year follow-up	Older group, 2-year follow-up	Younger group
MOCA	26.5 (1.5)	25.89 (1.48)	25 (2.17)	–
RAVLT-1	−0.13 (0.74)	0.04 (0.74)	0.01 (0.64)	0.11 (1.78)
RAVLT-5	−0.06 (0.9)	0.07 (0.99)	0.11 (1)	0.25 (0.83)
RAVLT-8	−0.39 (1.23)	−0.29 (1.25)	0.02 (1.13)	0.21 (0.83)
ROCF-copy	1.27 (0.51)	0.82 (0.54)	−0.34 (0.85)	–
ROCF-delayed recall	0.4 (0.75)	0.72 (0.84)	0.41 (0.79)	–
LM-immediate recall	0.78 (0.89)	0.99 (0.73)	1.11 (0.86)	–
LM-delayed recall	0.69 (0.99)	1 (0.87)	1.11 (0.93)	–
Phonemic verbal fluency	0.26 (0.99)	0.21 (0.91)	0.74 (0.94)	0 (1.17)
Semantic verbal fluency	0.57 (1.13)	0.25 (0.91)	0.12 (1.1)	0.32 (1.05)
Digit span	0.1 (1.01)	0.27 (0.87)	0.27 (0.97)	0.47 (0.77)
TMT-A	−0.01 (0.57)	0.16 (0.66)	0.44 (0.71)	–
TMT-B	0.54 (0.63)	0.67 (0.56)	0.67 (0.68)	–
General knowledge	1.33 (0.59)	1.38 (0.77)	1.51 (0.59)	–
Immediate memory score	0.32 (0.67)	0.52 (0.53)	0.56 (0.49)	–

*Results of neuropsychological assessment of older (n = 27) and younger adults (*n* = 25). Older adults completed the assessment three times: in a separate meeting shortly before the functional magnetic resonance imaging (fMRI) scan (baseline), 1 year following the baseline assessment (1-year follow-up) and 2 years following the baseline assessment (2-year follow-up). Only the results of the older adults who completed all the assessments are presented. Younger participants completed only a subset of the assessment after the fMRI scan. Details regarding subset of the older and younger adults can be found in Elkana et al. (2016) and Oren et al. (2015, 2017a). In all tests except the Montreal Cognitive Assessment (MoCA), scores are the age-specific standard scores (*mean* = 0, *SD* = 1). In the MoCA test, the score is the raw score (range, 0–30). The results indicate that performance in all tests was around the mean. Standard deviation is presented in parentheses. Immediate memory score is the average score of the first trail of the first word list of the Rey Auditory Verbal Learning Test (RAVLT) and immediate recall of logical memory test. Abbreviations: MoCA = Montreal Cognitive Assessment; RAVLT = Rey Auditory Verbal Learning Test, RAVLT-1 = 1st trial of the first word list, RAVLT-5 = 5th trial of the first word list, RAVLT-8 = delayed recall of the first word list; ROCF = Rey-Osterrieth complex figure test, copy and delayed recall; LM = logical memory test from the Wechsler Memory Scale (WMS); TMT = trail making test*.

### Experimental Design and Procedure

**Figure 1:**
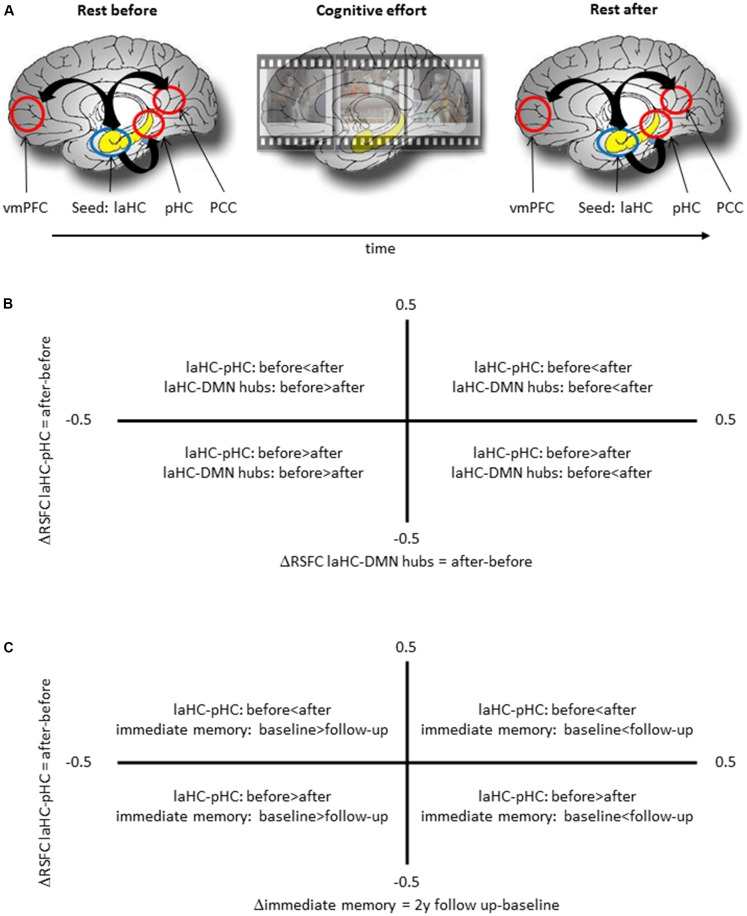
Schematic representation of the experimental procedure and the meaning of correlation between two deltas. **(A)** The experimental procedure included a run of rest, two runs of cognitive effort, including encoding of short movies and recognition questions, and another run of rest. **(B)** A positive correlation between ΔRSFC of left anterior hippocampus (laHC)–posterior hippocampi (pHC) and laHC–default mode network (DMN) hubs would mean that higher post-effort resting-state functional connectivity (RSFC) in the laHC–pHC connection is correlated with higher post-effort RSFC in the laHC–DMN hub connection. **(C)** A positive correlation between ΔRSFC of laHC–pHC and Δimmediate memory 2y would mean that lower post-effort RSFC in the laHC–pHC connection at baseline is associated with decline in immediate memory 2 years later.

All the participants completed an fMRI scan with five consecutive runs in the following order: resting state (378 s), task (582 s), anatomical scan (240 s), task (582 s), and resting state (378 s; Figure 1A). Between each run, participants were given about a minute to rest before the next run. The two resting-state runs would be termed “RSFC before” (effort) and “RSFC after” (effort), respectively.

In the resting-state runs, participants were instructed to lie still in the scanner with their eyes closed, not to think about anything in particular and not fall asleep. The task was an episodic memory task consisting of 16 consecutive cycles, each composed of three phases—encoding, distraction (mathematics), and recognition [a detailed description of the stimuli and paradigm may be found in Oren et al. (2016)]. In brief, a 21-s movie was played during the encoding phase. The movies in the task were unfamiliar, nonemotional, silent clips edited from feature-length films. The original films were not silent, but sound was omitted during editing. In order to make the encoding task more challenging, a distracting linguistic task was performed during encoding, in which participants had to indicate whether a string of letters was a word or pseudoword. Three words were presented during each movie: two words and one pseudoword or vice versa. The words appeared one at a time, like subtitles at the bottom of the screen, for 2 s each. Importantly, each word appeared 0.5 s before the frame that served as a target in a recognition question. The 9-s distraction phase followed immediately after the movie clip and consisted of three mathematical questions meant to diminish rehearsal and recency effects (Baddeley, 2003). The mathematical questions were composed of a simple equation (e.g., “56 + 7 =?”) at the top of the screen, with the correct answer and a foil in two separate rectangles. The recognition phase lasted 15 s and consisted of three recognition questions, in which the question “What did the last movie show?” was presented at the top of the screen with two picture choices presented below. The correct answer was a picture taken from the short movie that immediately preceded the question. The foil was a picture taken from the original feature-length film presenting the same characters and setting, but from a segment that did not appear in the experiment. A fixation cross of interchangeable length of 9 or 12 s was presented between the distraction and recognition phases, and between recognition and the next sequence. Each cycle of encoding, distraction, and recognition lasted between 63 and 69 s and presented different movies and questions. The paradigm was divided into two runs, and each run consisted of eight cycles. A run started with a fixation cross for 24 s and an additional single movie to habituate the neural response to the visual stimuli and to accustom the participants to the task. This movie was discarded from the analyses. Participants responded to the tasks using a handheld response box.

### MRI Acquisition

MRI scans were performed on a 3.0-Tesla MRI scanner (GE Signa EXCITE, Milwaukee, WI, USA) using an eight-channel head coil. Blood-oxygen-level-dependent (BOLD) functional MRI was acquired with T2*-weighted imaging: repetition time (TR) = 3,000 ms; echo time (TE) = 35 ms; flip angle (FA) = 90°; field of view (FOV) = 200 mm; matrix size = 96 × 96; 39 axial slices of 3-mm thickness, 0 gap. A high-resolution anatomical T1-weighted fast spoiled gradient echo imaging was acquired: FOV = 256 mm; matrix = 256 × 256; *TR* = 9.2 ms; *TE* = 3.5 ms; axial slices of 1-mm thickness, no gap. This anatomical scan was used for surface reconstruction. To minimize head movements, the participants’ heads were stabilized with foam padding. Stimuli were controlled using the PsychoPy software (Peirce, 2007, 2009) and presented *via* an liquid-crystal display (LCD) projector to a tilted (45°) mirror positioned over the participants’ foreheads. A MR-compatible response box was used to collect responses.

### Data Analysis

Data were analyzed using the BrainVoyager QX software (Formisano et al., 2005; Goebel et al., 2006) and in-house codes implemented in MATLAB. In the younger group, the resting-state data of one participant were discarded due to failure to complete the second run, and the fMRI task data of another three participants were discarded due to technical problems during the scan. In the older group, the resting-state data of one participant and the task data of the other five participants were discarded due to excessive head movements (see below). Additionally, a single task run of the other three older adults was also discarded due to head movements. Hence, the final RSFC analyses were performed on 24 younger and 27 older adults, and the final task analyses were performed on 22 younger and 23 older adults. Finally, head movements were excluded as potential confounding factors (see Supplementary Results).

### Preprocessing

Preprocessing of the functional scans included cropping of the first six TRs in each run to ensure that initial magnetization for each subsequent TR had reached a steady-state, 3D motion and slice scan time correction and linear trend removal. For the resting-state analyses, a low-pass filter was used (0.1 Hz). For the task analyses, a high-pass filter was used (one cycle per run). As mentioned above, runs in which head movements exceeded 3 mm were discarded from the analyses. Spatial smoothing was applied using a Gaussian spatial filter (6-mm full-width at half-maximum value). The functional images were superimposed on 2D anatomical images and incorporated into the 3D data sets through trilinear interpolation. The complete functional data set was transformed into Talairach space (Talairach and Tournoux, 1988). In the resting-state analyses, the average signals from the ventricles, white matter, and head movements were regressed out since they contained sources of spurious variance (Cole et al., 2010; Weinstein et al., 2016).

### Defining a Seed Region: the laHC

The aim of the study was to explore the RSFC of the HC and how it changed as a function of cognitive effort and age. Therefore, a seed-based RSFC analysis was employed with the HC as the seed. The HC was defined functionally based on activity level during the task that was performed between the two runs of rest, so it would have direct relevance to the cognitive effort. Based on previous studies (Spreng et al., 2010; Tromp et al., 2015), the HC was defined using a contrast that differentiated the activity level of the age groups during the encoding phase. To this end, the activation level was analyzed using a general linear model (combining the two task runs) which contained regressors for each phase, namely, encoding, mathematics, recognition, and a no-interest block. A whole-brain activation map was created for the contrast “younger vs. older during encoding” [*p* < 0.05, controlling for multiple comparisons across the whole brain was done using a false discovery rate (FDR) method; Benjamini and Hochberg, 1995; Benjamini and Yekutieli, 2001], cluster size >10 × 3^3^, with a gray matter mask (results of this activation analysis are presented in the Supplementary Results). The left aHC (laHC) emerged in this contrast with a lower activation level for older as compared to younger adults. This region was defined as a seed for the following RSFC analysis.

### Whole-Brain RSFC of the laHC

A whole-brain RSFC analysis was conducted with the laHC as the seed region. The time course, averaged across all the voxels in the seed, was extracted for each participant and for each rest run (i.e., rest before and after effort). A Pearson’s coefficient was used to calculate the correlation between the time course of the seed and each voxel across the whole brain. The resulting correlation coefficients were normalized using a Fisher transformation and entered into a random effects analysis of variance (ANOVA) with age (older/younger) and effort (before/after) as independent variables (*p* < 0.05, FDR-corrected, cluster >10 × 3^3^, with a gray matter mask).

### Defining Regions of Interest

Regions that demonstrated a significant effect in the whole-brain RSFC analysis were defined as regions of interest (ROIs) for all subsequent analyses. Specifically, regions that demonstrated a significant correlation with the laHC as a function of age (collapsed across rest before and after effort), effort (collapsed across age groups), or their interaction were defined as ROIs. In the case of bilateral homologous regions, we examined the correlation between them in order to determine whether they could be united into a single ROI for all subsequent analyses. To this end, the time course was extracted from both regions for each participant, and a Pearson correlation coefficient was calculated. The correlation coefficients of all the participants were Fisher transformed, and a one-sample *t*-test was used to determine whether they were significantly larger than zero. This was done separately for each rest run and group. In the case that homologous spatially separated ROIs were judged to be highly correlated, they were collapsed together by averaging the time course across all the voxels in the two ROIs, thus creating a united ROI.

### *Post Hoc* ROI Analyses

To further explain the nature of the effects that emerged from the whole-brain analysis, a *post hoc* analysis examined whether the connection between the seed and each ROI was significantly different from zero, separately for each age group. To this end, the following steps were taken. First, the time course of the ROI was extracted and averaged across all voxels in the ROI and both rest runs. Second, the correlation between the time courses of the seed and the ROI was calculated and normalized using a Fisher transformation. Third, across all participants in a given group, a one-sample *t-*test was used to determine whether the correlation was significantly different from zero (i.e., the seed and the ROI were functionally connected). This was a two-tailed test, as a correlation can be significantly higher or lower than zero. Controlling for multiple comparisons was done using the FDR method, separately for each group.

Next, we explored the possibility that differences in RSFC between older and younger adults emerged due to group differences in activation of the laHC. Dependency between seed activation and RSFC would be reflected by correlations between the two measures. Hence, we calculated these correlations to see if this interpretation was true. To this end, the time course, averaged across all the voxels in the seed, was extracted for each participant and task run. This time course was used to calculate percent signal change (%SC) from the baseline during encoding. Next, the correlation between %SC in the seed during encoding and Fisher-transformed r-value (correlation between the seed and an ROI, averaged across rest before and after effort) was calculated for each ROI, separately for each group. Controlling for multiple comparisons was done using the FDR method, separately for each group.

### Correlation Between Changes in RSFC Following Effort

Here we were interested to see whether changes in RSFC (difference in RSFC after effort as compared to before effort) between the laHC and different regions were related. This relationship was assessed as a Pearson correlation between two deltas (Figure 1B). To this end, a delta was calculated for each laHC–ROI connection, as the difference in RSFC after compared to before effort (ΔRSFC = RSFC after effort − RSFC before effort). Based on the literature (Salami et al., 2014; Jacobs et al., 2015), we focused on the correlation between the ΔRSFC of laHC–pHC and the ΔRSFC of laHC–DMN hubs. This correlation was calculated separately for each age group. Several control analyses were also conducted in order to determine the specificity of the results (see the “Results” section). Controlling for multiple comparisons across all the correlations calculated in this section was done using the FDR method.

### Correlation Between Change in RSFC and Future Cognitive Decline

This analysis focused on the relationship between ΔRSFC of laHC–pHC and subsequent changes in episodic memory with aging, assessed as the correlation between two deltas (Figure 1C). The first delta was the difference in RSFC before and after effort (ΔRSFC = RSFC after effort − RSFC before effort). The second delta was the difference in immediate memory score between follow-up and baseline (Δimmediate memory = immediate memory at follow-up − immediate memory at baseline). Immediate memory was measured as the average *z*-score of two neuropsychological tests for immediate episodic memory, namely, the first trial of the first word list of the RAVLT and immediate recall of logical memory test. These scores were used due to their sensitivity in detecting memory decline, and an average of two scores was used since a combination of measures provides better predictive accuracy than a single score (Bastin and Salmon, 2014; Gainotti et al., 2014). Note that this analysis used the difference in memory level between two time points (delta) and not memory level at a specific time point. This is due to the high education level of the older adults group, since high education level usually renders most neuropsychological tests insensitive to capture subtle decline (Elkana et al., 2016). Hence, comparing performance in the two time points should be more sensitive in the current study group. A different correlation was constructed for each relevant connection and for each year. Specifically, one correlation used the Δimmediate memory between the scores of the 1-year follow-up and baseline (to be termed Δimmediate memory 1y) and another Δimmediate memory of the 2-year follow-up and baseline (to be termed Δimmediate memory 2y). Additional control analyses were conducted (see the “Results” section). Controlling for multiple comparisons across all the correlation calculated in this section was done using the FDR method.

## Results

### Whole-Brain Resting-State Functional Connectivity of the laHC

**Figure 2:**
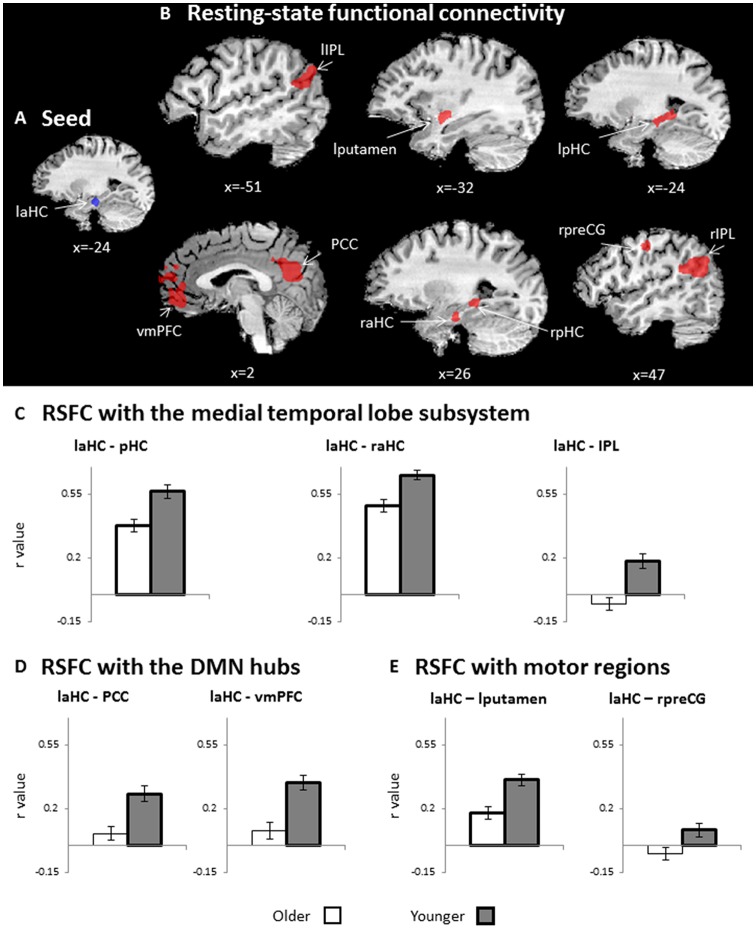
RSFC. **(A)** Seed region for RSFC: laHC. **(B)** Whole-brain maps depicting lower RSFC in older as compared to younger adults (*n* = 51, random effects, *p* < 0.05, false discovery rate (FDR)-corrected, cluster >10 × 3^3^). For visualization purposes only, **(C–E)** bar graphs representing *r* values (RSFC, collapsed across rest before and after effort) that emerged in the whole-brain RSFC analysis, as depicted in **(B)**. Since **(C–E)** are taken from RSFCs that show a significant group effect (depicted in **B**), all RSFCs are significantly lower for older (white) than for younger (dark gray) adults. Regions of interest (ROIs) were divided into three groups: **(C)** medial temporal lobe (MTL) subsystem, **(D)** midline DMN hubs, and **(E)** motor regions. Error bars represent standard errors. Bars denoted with thick borders indicate significant difference from zero. Note that although statistical analyses were conducted on Fisher-transformed correlations, the results are presented in the original correlation for the reader’s convenience. Error bars represent standard errors.Abbreviations: l, left; r, right; aHC, anterior hippocampus; pHC, posterior hippocampus, IPL, inferior parietal lobule; vmPFC, ventromedial prefrontal cortex; PCC, posterior cingulate cortex; preCG, precentral gyrus.

The first aim of the study was to examine whether the connections of the laHC at rest change as a function of age and/or cognitive effort. To this end, a whole-brain RSFC analysis was conducted, using the laHC as the seed region (Figure 2A) and age (older/younger) and effort (before/after) as independent variables. The only effect that emerged was that of age (*p* < 0.05, FDR-corrected, cluster >10 × ^3^; Figure 2B and Table 2). For all the connections with the seed, RSFC was lower for older than for younger adults. The regions that demonstrated this effect could be divided into three groups. The first group was composed of regions of the MTL subsystem of the DMN: right aHC (raHC), left pHC (lpHC), right pHC (rpHC), and bilateral IPL. The second group consisted of the two midline DMN hubs: PCC and vmPFC. Motor regions such as the right precentral gyrus (preCG) and the left putamen constituted the third group. Notably, no main effect for effort or an interaction between age and effort was found.

**Table 2:** Main effect for age in the resting-state functional connectivity (RSFC) analysis with the left anterior hippocampus (laHC) as seed.

Region	Talairach *x, y, z*	*F*_(1,49)_	*p*-value	*N* of voxels
DMN’s MTL subsystem
rpHC	26, −35, −9	16	0.000213	403
raHC	29, −17, −18	21.81035	0.000024	381
lpHC	−25, −38, −6	25.88377	0.000006	1647
right IPL	50, −56, 18	37.79749	0	5429
left IPL	−52, −65, 21	23.88179	0.000011	2997
DMN’s hubs
vmPFC	2, 46, −9	40.16652	0	8137
dorsal PCC	5, −57, 18	39.62678	0	9250
Motor regions
preCG	47, −11, 43	21.52127	0.000026	353
left putamen	−31, −8, −6	25.88567	0.000006	756

*Results of RSFC analysis, with the laHC as a seed. These regions showed main effect for group, in which RSFC was lower for older than in younger adults. The regions are divided according to default mode networks (DMN’s) subsystems (Andrews-Hanna et al., 2010) and additional motor regions. Random effect, *p* < 0.05, false discovery rate (FDR)-corrected, cluster >10 × 3^3^*.

### Defining ROIs

Regions that emerged in the whole-brain RSFC analysis were defined as ROIs. Accordingly, regions that demonstrated main effect for age (i.e., differences between older and younger adults, collapsed across rest before and after effort), which was the only significant effect, were defined as ROIs. The set included two pairs of bilateral homologous regions: pHC and IPL. In order to confirm that each homologous pair could be averaged together for subsequent analyses, we examined the correlation between them. The pHC were highly correlated at rest, both before and after cognitive effort, for younger (rest before: *r*_(24)_ = 0.4, *p* = 0.05; rest after: *r*_(24)_ = 0.57, *p* = 0.004) and older (rest before: *r*_(27)_ = 0.51, *p* = 0.006; rest after: *r*_(27)_ = 0.7, *p* = 3.8*10^−5^) adults; thus, they were collapsed together. As a control analysis, we examined the correlation between the raHC and the posterior sections of both HC, but found no significant correlation. Namely, the raHC was not correlated with the rpHC at rest, neither before nor after cognitive effort, for younger (rest before: *r*_(24)_ = 0.22, *p* = 0.3; rest after: *r*_(24)_ = 0.31, *p* = 0.14) or older (rest before: *r*_(27)_ = 0.36, *p* = 0.06; rest after: *r*_(27)_ = -0.27, *p* = 0.17) adults. Similarly, the raHC was not correlated with the lpHC at rest before or after effort for younger (rest before: *r*_(24)_ = 0.25, *p* = 0.23; rest after: *r*_(24)_ = 0.16, *p* = 0.45) or older (rest before: *r*_(27)_ = 0.36, *p* = 0.06; rest after: *r*_(27)_ = 0.08, *p* = 0.69) adults. Hence, only the posterior sections of both HC were collapsed together. With regard to the IPL, the left and right IPL were highly correlated at rest before and after effort for younger (rest before: *r*_(24)_ = 0.66, *p* = 0.0003; rest after: *r*_(24)_ = 0.8, *p* = 3.4 × 10^−7^) and older (rest before: *r*_(27)_ = 0.63, *p* = 0.0003; rest after: *r*_(27)_ = 0.37, *p* = 0.05) adults, and so were also collapsed together for subsequent analyses. Hence, the final set of ROIs included seven regions: pHC (bilateral), raHC, IPL (bilateral), PCC, vmPFC, right preCS, and left putamen.

### *Post Hoc* ROI Analyses

Lower RSFC for older as compared to younger adults may be due to negative connections between the seed and ROIs or a lack of connection (i.e., RSFC not different than zero) in older adults. Hence, to further understand the age-related changes, *post hoc* analyses were conducted to examine whether the connections between the seed and each ROI was significantly different from zero (i.e., significantly higher or lower than zero). The tests were performed separately for each group and averaged across both rest runs (i.e., before and after effort) due to lack of effort effect. In the younger group, all connections were significantly higher than zero (*p* < 0.05, FDR-corrected; Figures 2C–E, thick borders). In contrast, in the older adults, the coupling of the laHC with the PCC, vmPFC, IPL, and right preCG were not significantly different from zero (*p* > 0.05, corrected; Figures 2C–E, thin borders). The coupling of the laHC with the pHC, raHC, and left putamen were significantly higher than zero (*p* < 0.05, corrected; Figures 2C–E, thick borders). Thus, in the older adults, the laHC was functionally unconnected to the cortical regions, which showed RSFC in the younger adults, while the subcortical connections were lower than those of younger adults.

One may claim that the lower RSFC for older adults that emerged in our study was due to the lower seed activation in the older adults group. If that was the case, then one would also expect a significant correlation between laHC activation during encoding and RSFC between the laHC and each ROI. To examine this possibility, we calculated these correlations but did not find a significant correlation in the older or the younger adults (*p* > 0.05, FDR-corrected, for all correlations). Hence, we conclude that it is less likely that the group differences in RSFC were derived from group differences in the activation of the laHC during encoding.

### Correlation Between Changes in RSFC Following Effort

We aimed to test whether change in RSFC of one connection is associated with change in RSFC of another connection (i.e., RSFC of the laHC with a certain ROI and with another ROI). Change in RSFC is the difference between RSFC after and before effort. Hence, we looked at the correlation between two deltas (ΔRSFC) and whether they vary with age.

**Figure 3:**
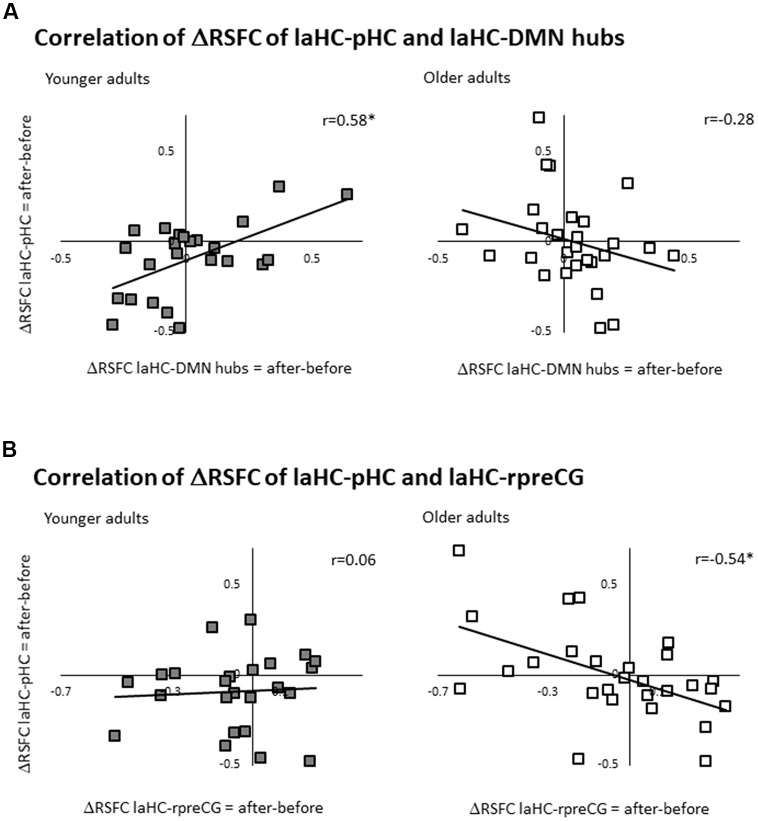
Correlations between ΔRSFCs. Scatter plots representing the linear relationship between two ΔRSFCs (RSFC after effort − RSFC before effort) of two connections. **(A)** For younger adults only, higher post-effort RSFC in the laHC–pHC connection was associated with higher post-effort RSFC in the laHC–DMN hub connection. **(B)** For older adults only, higher post-effort RSFC in the laHC–pHC connection was associated with lower post-effort RSFC in the laHC–right preCG connection. Note that although statistical analyses were conducted on Fisher-transformed correlations, the results are presented in the original correlation for the reader’s convenience. Abbreviations: l, left; r, right; aHC, anterior hippocampus; pHC, posterior hippocampus; DMN, default mode network; preCG, precentral gyrus; *p < 0.05, FDR-corrected.

Here, the ΔRSFC (ΔRSFC = RSFC after effort − RSFC before) for the connection of the laHC with each ROI was calculated. Based on the literature, we focused on the correlation between the ΔRSFC laHC–pHC connection and the ΔRSFC laHC–DMN hub connection (i.e., averaging all the voxels from the PCC and the vmPFC together, thus creating a united ROI of the DMN hubs, see validation below; this correlation would be termed below as the “main correlation”). This main correlation was calculated separately for each group. For the younger group, there was a positive correlation (*r*_(22)_ = 0.58, *p* = 0.036, FDR-corrected; Figure 3A). Interestingly, for older adults, the correlation was non-significant (*r*_(25)_ = −0.28, *p* = 0.3, FDR-corrected; Figure 3A). The difference between the two correlations was significant (*z* = 3.17, *p* = 0.001, two-tailed), as determined by comparing the independent correlation coefficients (Lowry, 2016). This means that only for younger adults, post-effort increase in the connection between the laHC and the pHC was related to a similar increase in the connection between the laHC and the DMN hubs, and vice versa. This relationship was not present with older age.

Several control analyses were conducted to examine whether the pattern that emerged in the main correlation (i.e., the correlation between ΔRSFC of laHC–pHC and laHC–DMN hubs) was also evident in other connections. The first control analysis examined whether the main correlation emerged mainly in the anterior–posterior intra-HC connection, or whether the left–right intra-HC connection could also show the same correlation with the DMN hubs. To this end, the correlation between ΔRSFC of laHC–raHC and laHC–DMN hubs was calculated. Results showed that although the correlations were similar, they were non-significant (younger: *r*_(22)_ = 0.22, *p* = 0.4, FDR-corrected; older: *r*_(27)_ = −0.25, *p* = 0.4, FDR-corrected). This indicates that the main correlation emerged primarily in the anterior–posterior intra-HC connection.

Next, we examined whether the main correlation changed if it was calculated with each DMN hub separately, thus establishing the validity of collapsing together the PCC and vmPFC. Here, we found that when separating the hubs, the correlation between the deltas became marginally positive for younger adults following correction for multiple comparisons and remained non-significant for older adults. This was true for the correlation between ΔRSFC of laHC–pHC and laHC–PCC (younger: *r*_(22)_ = 0.48 *p* = 0.06, FDR-corrected; older: *r*_(27)_ = −0.34, *p* = 0.18, FDR-corrected) and also for the correlation between ΔRSFC of laHC–pHC and laHC–vmPFC (younger: *r*_(22)_ = 0.45, *p* = 0.06, FDR-corrected; older: *r*_(27)_ = −0.09, *p* = 0.6, FDR-corrected). These results indicate that the hubs had similar RSFC patterns, so they were collapsed together to increase statistical power.

We also checked whether the main correlation emerged mainly in the connection to the DMN hubs, as compared to other DMN cortical regions. To this end, the correlation between ΔRSFC of laHC–pHC and laHC–IPL was calculated. The results were non-significant (younger: *r*_(22)_ = 0.27, *p* = 0.3, FDR-corrected; older: *r*_(27)_ = −0.25, *p* = 0.3, FDR-corrected), suggesting that the main correlation emerged primarily for the hubs of the DMN.

Then, we tested the correlation to other regions that are not part of the DMN. Namely, the correlation was recalculated, once with the left putamen and once with the right preCG. For the left putamen, there were non-significant correlations for both groups (correlation between ΔRSFC of laHC–pHC and laHC–left putamen, younger: *r*_(22)_ = 0.16, *p* = 0.5, FDR-corrected; older: *r*_(27)_ = 0.46, *p* = 0.06, FDR-corrected). Surprisingly however, for the right preCG, there was a significant correlation for the older but not the younger adults (correlation between ΔRSFC of laHC–pHC and laHC–right preCG, younger: *r*_(22)_ = 0.06, *p* = 0.7, FDR-corrected; older: *r*_(27)_ = −0.54, *p* = 0.036, FDR-corrected; Figure 3B). The difference between the correlation of the older and younger adult was significant (*z* = 2.21, *p* = 0.021, two-tailed). These results indicate that the correlation between the HC and DMN hubs that was seen in younger adults was not present in older adults and that a different correlation was seen between the HC and cortical motor region (preCG).

The final control analysis examined whether the main correlation emerged mainly in RSFC, as compared to FC during encoding. To answer this question, FC during encoding was calculated and correlations between FC of the different regions were calculated, separately for each group. None of the results were significant. Specifically, the correlations between FC of laHC–pHC and laHC–DMN hubs were non-significant (younger: *r*_(20)_ = 0.08, *p* = 0.7, FDR-corrected; older: *r*_(21)_ = 0.31, *p* = 0.2, FDR-corrected). Similarly, the correlations between FC of laHC–pHC and laHC–right preCG were non-significant (younger: *r*_(20)_ = 0.39, *p* = 0.17, FDR-corrected; older: *r*_(21)_ = 0.48, *p* = 0.06, FDR-corrected). These results suggest that in the current study, the correlations between the HC and cortical regions emerged with regard to changes in RSFC following effort.

Together, the results show that for younger adults, increased intra-HC RSFC following effort was associated with an increased RSFC of the HC with DMN hubs. Older adults did not show this pattern. For them, increased intra-HC RSFC following effort was associated with decreased RSFC of the HC and right preCG. This means that older age changed the regions that participated in the correlation, as well as the direction of the correlation.

### Correlation Between Change in RSFC and Future Memory Decline

**Figure 4:**
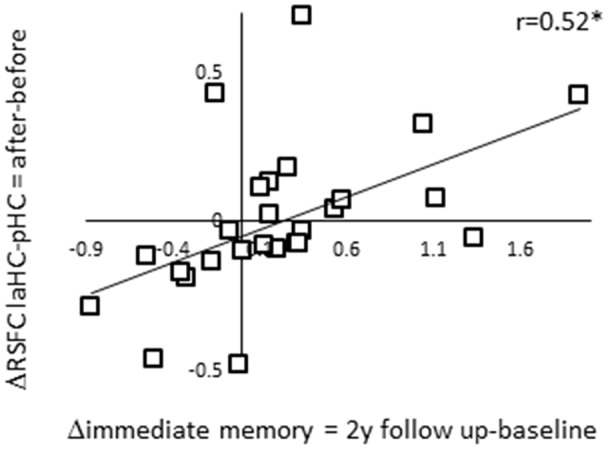
Correlation between ΔRSFC and Δimmediate episodic memory score of older adults at the 2-year follow-up. A scatter plot representing the linear relationship between ΔRSFC (RSFC after effort − RSFC before effort) and Δimmediate memory 2y score (immediate memory 2y follow-up − immediate memory baseline). Lower post-effort RSFC in the laHC–pHC connection was associated with lower scores for immediate memory at the 2-year follow-up. Note that although statistical analyses were conducted on Fisher-transformed correlations, the results are presented in the original correlation, for the reader’s convenience. Abbreviation: l, left; aHC, anterior hippocampus; pHC, posterior hippocampus; y, year; *p < 0.05, FDR-corrected.

Thus far, we showed that the RSFC of the laHC decreased with age. We also showed that ΔRSFC of laHC–pHC was related to ΔRSFC of laHC with other regions and that the regions and direction of the correlations changed with age. We were next interested to examine the ability of ΔRSFC of laHC–pHC to predict future decline in episodic memory in older adults. To this end, we calculated the correlations between ΔRSFC and Δimmediate memory scores (ΔRSFC = RSFC after effort − RSFC before effort; Δimmediate memory 1y = immediate memory at 1-year follow-up − immediate memory at baseline; Δimmediate memory 2y = immediate memory at 2-year follow-up − immediate memory at baseline). The correlation between ΔRSFC of laHC–pHC and Δimmediate memory 2y was significant (*r*_(24)_ = 0.52, *p* = 0.045, FDR-corrected; Figure 4). The correlation between ΔRSFC of laHC–pHC and Δimmediate memory 1y was non-significant (*r*_(24)_ = 0.32, *p* = 0.2, FDR-corrected). Hence, for older adults, decrease in the RSFC of the laHC–pHC following effort was associated with a decrease in immediate memory 2 years after.

Control analyses examined the uniqueness of the correlation described above by calculating the correlations between ΔRSFC of the HC with other ROIs and Δimmediate memory 2y. The first control analysis examined whether the prediction of future decline in immediate episodic memory was specific to the anterior–posterior intra-HC connection, as opposed to the left–right intra-HC connection. To this end, the correlation between ΔRSFC of laHC–raHC and Δimmediate memory 2y was calculated but was non-significant (*r*_(24)_ = 0.36, *p* = 0.17, FDR-corrected). The following analysis examined the predictability of the two ΔRSFC of connections that emerged in the previous analysis, namely, laHC–DMN hubs and laHC–right preCG. Yet, in both of these cases, the correlation was non-significant (correlation between ΔRSFC of laHC–DMN hubs and Δimmediate memory 2y *r*_(24)_ = −0.18, *p* = 0.4, FDR-corrected; correlation between ΔRSFC of laHC–right preCG and Δimmediate memory 2y *r*_(24)_ = −0.29, *p* = 0.2, FDR-corrected). These results establish the ΔRSFC of the laHC–pHC to predict future memory decline. Thus, older adults who were negatively affected by the effort, measured as reduced RSFC between anterior and pHC following an effortful task, demonstrated deterioration in immediate episodic memory in the subsequent 2 years. Those older adults who maintained high RSFC levels after effort also maintained their learning abilities, as indicated by their improved performance on follow-up cognitive testing.

## Discussion

The current study examined how the RSFC of the laHC changes with cognitive effort and age, and the ability of effort-related changes in RSFC to predict future memory decline with aging (Figure 1). Here, cognitive effort was composed of mnemonic (encoding and recognition) and nonmnemonic (mathematics) tasks. The results showed that at rest, the laHC of older adults demonstrated lower subcortical connections compared to younger adults (Figure 2C; i.e., RSFC significantly higher than zero, but lower than that of younger adults), and that at the group level, the laHC of older adults was actually unconnected to cortical regions (Figures 2C–E; i.e., RSFC not significantly higher than zero). For younger adults, post-effort increase in RSFC of the laHC with the pHC was associated with post-effort increase in RSFC of the laHC with DMN hubs (Figure 3A). Older adults showed a different pattern, where post-effort increase in RSFC of the laHC with the pHC was related to post-effort decrease in RSFC of the laHC with the right preCG (Figure 3B). Finally, decreased RSFC of the laHC with the pHC after cognitive effort in older adults was associated with memory decline 2 years later (Figure 4). Notably, our findings refer to RSFC, i.e., synchronization in the BOLD signal of spatially distinct regions, as opposed to anatomical connectivity.

The laHC had lower activation (Figure 2A) and RSFC (Figures 2C–E) in older adults, corroborating previous findings of pronounced age-related decline in the aHC in terms of anatomy, activation (Spreng et al., 2010; Ta et al., 2012; Tromp et al., 2015), and RSFC (Salami et al., 2016). In younger adults, the aHC is functionally connected at rest with the PCC, vmPFC, IPL, and other frontal and temporal regions (Vincent et al., 2006). Compatible with these findings, we showed that in younger adults, the laHC was functionally connected to three sets of regions (Figure 2; Table 2). The first set included regions from the DMN MTL subsystem, namely, other sections of the HC, as well as bilateral IPL (Andrews-Hanna et al., 2010). The second set included the PCC and vmPFC, which are midline DMN hubs (Buckner et al., 2009). The laHC was further correlated with motor regions, i.e., left putamen and right preCG, in keeping with findings that the MTL subsystem can correlate with regions outside the DMN (Campbell et al., 2013). For older adults, the laHC maintained to a lesser degree its subcortical connections (Figure 2C), but connections with the cortical regions (Figures 2D,E), including regions of the DMN, were not observed. These findings provide additional evidence of disrupted RSFC of the DMN at older age (Ferreira and Busatto, 2013).

Cognitive effort was associated with an increase in RSFC of the laHC with both pHC and DMN hubs, but only for younger adults (Figure 3A). This finding beautifully matches and expands previous studies that showed that the RSFC of the DMN increases following encoding in younger but not in older adults (Jacobs et al., 2015) and that in younger age there is a correlation between RSFC of the HC and DMN cortical regions (Salami et al., 2014). Hence, intra-HC and inter-HC connections change in a similar fashion following cognitive effort in younger age. The DMN hubs are implicated in episodic memory (van Kesteren et al., 2010; Shapira-Lichter et al., 2013; Oren et al., 2017b), and the connection between them and the HC is important for memory function (Moscovitch et al., 2016). The HC is thought to be a fast learning system that binds features into an event and spreads its representation to the cortical DMN, which integrates events over time (Preston and Eichenbaum, 2013; O’Reilly et al., 2014). Thus, it is not surprising to find that after cognitive effort that includes encoding and retrieval, the HC will be connected more strongly to DMN hubs. Lack of such an association in older age can be regarded as yet another manifestation of declining memory function with age and is in line with a similar previous null result in older adults (Jacobs et al., 2015). Yet, older age is not only associated with losses but also with the additions that are not found in younger adults (Park and Reuter-Lorenz, 2009; Oren et al., 2018). We found that only for older adults, when cognitive effort was associated with increased intra-HC RSFC, it was also associated with decreased RSFC between the laHC and right preCG (Figure 3B). The preCG is implicated with regard to memory, as it is activated in tasks of encoding or retrieval of episodic memory (Stolz et al., 2012; Brod et al., 2015) and is involved at encoding in patients with mild cognitive impairment (Yogev-Seligmann et al., 2016). Future studies may shed light on the functional significance of the age-related changes we observed, and whether they may reflect cognitive deficits in older adults, compensation, or dedifferentiation (Grady, 2012).

The aHC–pHC connection is central in our findings. The anterior and posterior sections contribute to complementary aspects of memory (Poppenk et al., 2013; Strange et al., 2014; Moscovitch et al., 2016). Thus, the aHC–pHC connection may create an integrated representation of an event, which can then be transformed to the cortex (Moscovitch et al., 2016). The fact that at older age a decrease in this connection following cognitive effort is related to memory decline is in keeping with this idea (Figure 4). The converse of this finding is that older adults who had higher intra-HC RSFC after cognitive effort, similar to the pattern seen in younger adults (Jacobs et al., 2015), benefited from the repeated presentation of the same tests in successive years and improved on these tests over time. In this analysis, we used the difference between performance in memory tests in two time points, rather than performance in a certain time point, to overcome the low sensitivity of neuropsychological tests in our highly educated population (Elkana et al., 2016). The correlation between changes in RSFC and changes in memory level was significant only after 2 years between baseline and follow-up neuropsychological assessments, but not after the first year. Though a null result should be interpreted with caution, we suspect that it arises from the fact that a gap of a single year between assessments is not sufficient to give rise to substantial changes in memory level in healthy older adults (see Table 1 for smaller difference between baseline and 1y follow-up compared to baseline and 2y follow-up). The fact that the two correlations were in the same direction and of similar magnitude implies a trend that gains momentum over time.

The age differences in the effect of cognitive effort on RSFC patterns may be the result of neural networks that were engaged during the cognitive effort period or differences in task performance. We found that during encoding, younger adults significantly deactivated regions from the DMN, but we did not find this pattern in the older adults (Supplementary Results). Moreover, older adults demonstrated activation in regions that were not seen in the younger adults, which may suggest some kind of compensation. Hence, the different networks that emerged during encoding in each group may have contributed to the observed age differences in the RSFC. Additionally, we previously showed that during the cognitive effort, performance of the older adults was less accurate and slower, compared to the younger adults (Oren et al., 2016, 2017b, 2018). Hence, the older adults found the tasks more difficult, which can explain the age differences in activation (Supplementary Results) and in RSFC.

One obstacle in comparing the BOLD signal of older and younger adults is that this signal reflects interaction of many factors, all of which are affected by older age (D’Esposito et al., 2003; Wright and Wise, 2018). These factors include neuronal activity but also cerebral blood flow, cerebrovascular reactivity, and cerebral metabolic rate of oxygen. Hence, it is difficult to know whether neuronal or rather non-neural factors underlie the differences between the age groups in the BOLD signal. Nevertheless, this problem does not apply to within-group results, such as the finding that in older adults, changes in RSFC are associated with future memory decline (Figure 4).

To conclude, in the current study, we focused on age-related changes in intra-HC RSFC along the long axis following cognitive effort that involves both mnemonic and nonmnemonic tasks. Post-effort increase in intra-HC RSFC was associated with greater connection of the HC and DMN hubs in younger adults, a connection which is thought to be an essential stage in memory formation. The association between intra-HC and DMN hubs did not appear in older age. Older adults had a negative association between changes in intra-HC and changes in HC-right preCG RSFC, unlike younger adults. Finally, intra-HC RSFC was associated with future memory change in older age, so that post-effort decrease in intra-HC RSFC was associated with decreased memory function two years later. Lack of a similar association to memory change 1 year later may be due to minimal change in memory function of healthy older adults during a 1-year period or difficulty of the neuropsychological tests to detect such a change if it does exist. Overall, our findings show that modulation of intra-HC RSFC by cognitive effort is changed with age and that this change is associated with alterations in memory level in healthy, cognitively intact older adults. Future studies should be performed to advance these findings and develop interventions that will help to delay progression of decline.

## Ethics Statement

The experimental procedures were approved by the Tel Aviv Sourasky Medical Center Institutional Review Board and all participants provided written informed consent.

## Author Contributions

Conceptualization was done by NO, EA, and IS-L. NO and YL contributed to the methodology. EA and YL contributed to the resources. NO contributed to the fMRI analysis and wrote the manuscript. OR-E, OE, and LC conducted and analyzed the follow-up assessments. YL, EA, and OE were in charge of supervision.

## Conflict of Interest Statement

The authors declare that the research was conducted in the absence of any commercial or financial relationships that could be construed as a potential conflict of interest.
