# Supplementary material for: Changes in Resting-State Functional Connectivity of the Hippocampus Following Cognitive Effort Predict Memory Decline at Older Age—A Longitudinal fMRI Study
Source: Front Aging Neurosci. 2019 Jul 16;11:163. doi: 10.3389/fnagi.2019.00163 (PMC6660259; doi:10.3389/fnagi.2019.00163)
Supplement: Supplementary file 1 [file Table_1.docx]

**Supplementary Results**

**Age differences in activation during encoding**

Examining the whole brain activation results can show which neural networks older and younger adults engaged differently during the period between the resting-state runs. A whole brain activation map was created for the contrast 'younger vs. older during encoding' (p < 0.05, controlling for multiple comparisons across the whole brain was done using an FDR method (Benjamini and Hochberg, 1995; Benjamini and Yekutieli, 2001), cluster size > 10*3^3^, with a grey matter mask). This contrast yielded 12 regions (Figure S1 A; Table S1). Notably, there was no substantial overlap between the regions that emerged in this activation analysis and the regions that emerged in the resting-state functional connectivity (RSFC) analysis (compare Figure 2 B to Figure S1 A).

To examine whether the activations that emerged in the whole brain analysis were different zero (i.e., baseline), a post hoc ROI analysis was applied for each region and group. To this end, for each participant and region, a time course was extracted, averaged across all the voxels in that region. Next, percent signal change (%SC) from baseline was calculated for the activation periods. Finally, for each region and group, a 1-sample t-test (2-tailed) examined whether the %SC was significantly different from zero. Results are presented in Figure S1 B and in Table S1.

Only two regions had higher activation for younger than for older adults: left anterior hippocampus (laHC, the seed region for RSFC analysis), and right parietal-occipital region (Figure S1, blue). The other regions had higher activation (or less de-activation) for older than younger adults (Figure S1, red): right and left lateral temporal cortex (LTC), right and left superior precuneus, cuneus, anterior and inferior ventromedial prefrontal cortex (vmPFC), paracentral lobule, left orbitofrontal cortex, and left supramarginal gyrus. More specifically, as indicated by Table S1, for the older adults, activation during encoding was not different from baseline (staring at a fixation cross) in the laHC, anterior and inferior vmPFC and bilateral LTC. These are regions of the default mode network (DMN; (Andrews-Hanna et al., 2010)) and all except the LTC are directly related to episodic memory (Andrews-Hanna et al., 2014; Moscovitch et al., 2016). This result is in line with reports about lesser engagement of the DMN in older age (Hafkemeijer et al., 2012). In contrast to the older adults, younger adults' activations were not different from baseline in the left superior precuneus, left orbitofrontal cortex and paracentral lobule. This means that during encoding, the older adults activated regions that were not activated by the younger adults, perhaps as a mean of compensation (Grady, 2012).

**
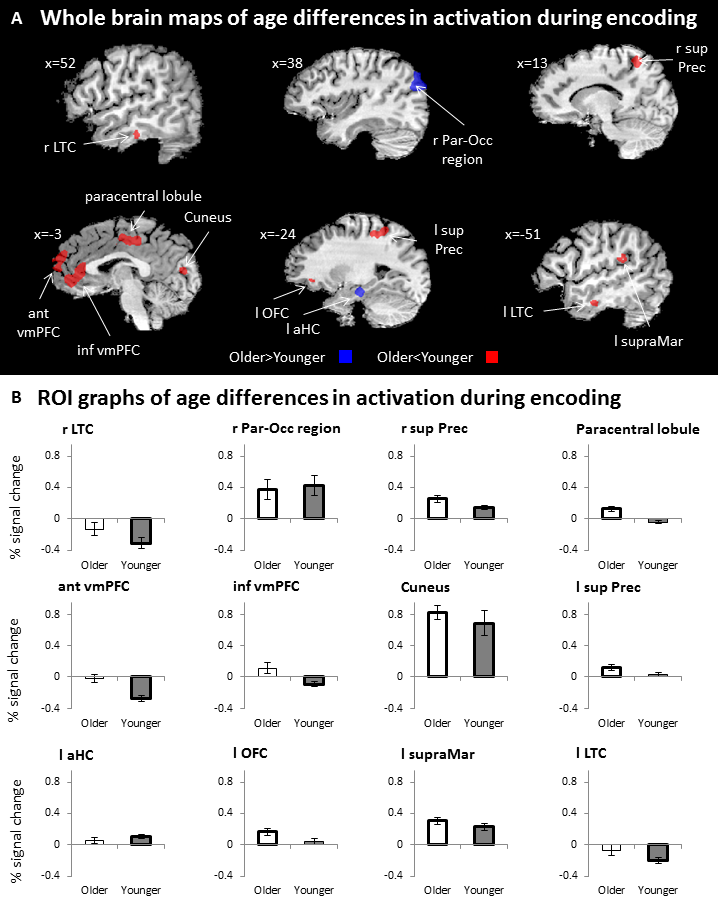
**

**Figure S1. Age differences in activation during encoding**. (A) Whole brain activation analysis maps presenting regions that during encoding were more activated for older (blue) or younger (red) adults (n = 45, random effects, p < 0.05, FDR corrected, cluster > 10*3^3^). (B) For visualization purposes only, bar graphs representing percent signal change (%SC) that emerged in the whole-brain activation analysis, as depicted in (A). Post hoc analysis for each region and group examined whether activation was significantly different from zero. Bars denoted with thicker borders indicate significant difference from zero. Error bars represent standard errors. Abbreviations: l = left, r = right, LTC = lateral temporal cortex; Par-Occ = parietal-occipital; sup Prec = superior precuneus; ant = anterior; vmPFC = ventromedial prefrontal cortex; inf = inferior; aHC = anterior hippocampus, OFC = orbitofrontal cortex; supraMar = supramarginal.

**Table S2. Percent signal change in each region and group and age differences.**

| **Region** | **percent signal change older adults** | **percent signal change younger adults** | **Age differences** |
| --- | --- | --- | --- |
| left anterior hippocampus | %SC ~= baseline | %SC > baseline | older < younger |
| right parietal-occipital region | %SC > baseline | %SC > baseline | older < younger |
| right lateral temporal cortex | %SC ~= baseline | %SC < baseline | older > younger |
| left lateral temporal cortex | %SC ~= baseline | %SC < baseline | older > younger |
| right superior percuneus | %SC > baseline | %SC > baseline | older > younger |
| left superior percuneus | %SC > baseline | %SC ~= baseline | older > younger |
| cuneus | %SC > baseline | %SC > baseline | older > younger |
| anterior ventromedial prefrontal cortex | %SC ~= baseline | %SC < baseline | older > younger |
| inferior ventromedial prefrontal cortex | %SC ~= baseline | %SC < baseline | older > younger |
| paracentral lobule | %SC > baseline | %SC ~= baseline | older > younger |
| left orbitofrontal cortex | %SC > baseline | %SC ~= baseline | older > younger |
| left supramarginal gyrus | %SC > baseline | %SC > baseline | older > younger |

**Gender as a Possible Confounder**

Since the proportion of females was different in the two age-groups (57% females in the older group and 36% females in the younger group), we examined if the differences between the age groups may be related to gender. Therefore, the measure of RSFC between the seed (left anterior hippocampus; laHC) and each of the ROIs that emerged in the whole brain RSFC analysis (see section 3.1 in the manuscript) was re-analyzed. For each connection between seed and ROI, Fisher transformed correlation coefficients of rest before and after effort were collapsed together. The difference between older and younger adults was examined separately for males and for females, using a 2-tailed independent-sample t-test.

The data indicated that the differences between the groups were similar for both males and females in all the connections (Figure S2). Statistical analyses indicated that the older group had significant lower RSFC than the younger group in all the connections, both for males and for females (Table S2). These results supported the idea that the differences in RSFC between older and younger adults were not due to the difference in proportion of males and females in each group.


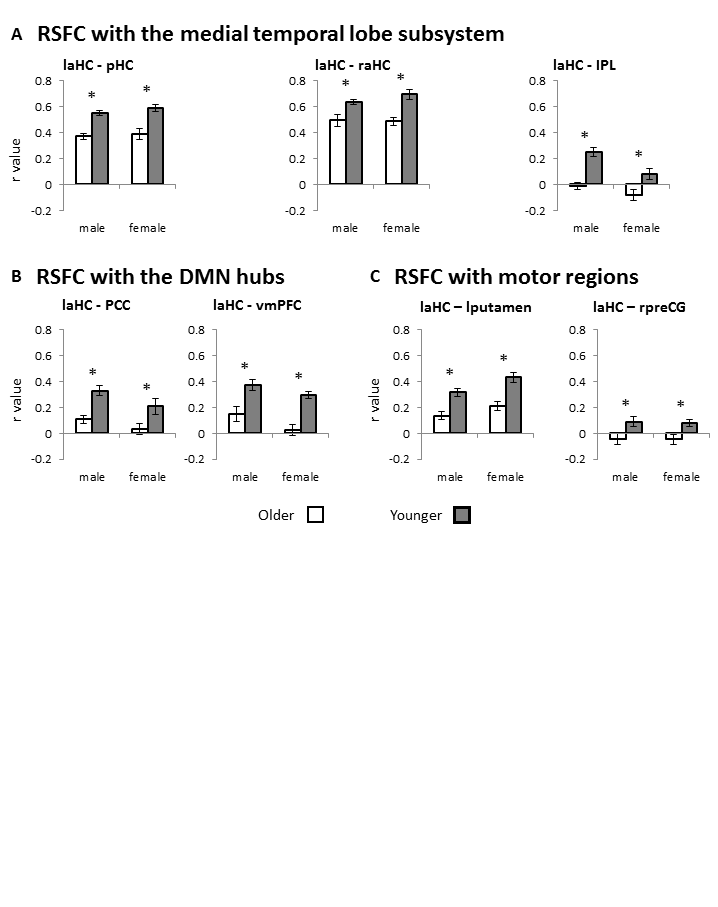


**Figure S2.** **Resting-state functional connectivity**. (A-C) Bar graphs representing r values (RSFC, collapsed across rest before and after effort) that emerged in the whole-brain RSFC analysis (as depicted in Figure 2 in the manuscript), separately for males and females. RSFC was lower for older than younger adults in all ROIs for both males and females. ROIs were divided into three groups: (A) MTL subsystem, (B) midline DMN hubs, and (C) motor regions. Error bars represent standard errors. Note that although statistical analyses were conducted on Fisher-transformed correlations, the results are presented in the original correlation for the reader's convenience. Error bars represent standard errors. Abbreviations: l=left, r=right, aHC=anterior hippocampus, pHC=posterior hippocampus, IPL=inferior parietal lobule, vmPFC=ventromedial prefrontal cortex, PCC= posterior cingulate cortex; preCG=precentral gyrus, * p<0.05.

**Table S2. Differences between older and younger adults separately for males and females.**

| **RSFC** | **Males** | **Females** |
| --- | --- | --- |
| laHC – pHC | t_(25)_ = 5.7, p < 0.001 | t_(22)_ = 3.4, p = 0.002 |
| laHC – raHC | t_(25)_ = 2.28, p = 0.009 | t_(22)_ = 3.89, p = 0.001 |
| laHC – IPL | t_(25)_ = 5.4, p < 0.001 | t_(22)_ = 2.54, p = 0.019 |
| laHC – PCC | t_(25)_ = 4.01, p < 0.001 | t_(22)_ = 2.27, p = 0.033 |
| laHC – vmPFC | t_(25)_ = 2.95, p = 0.007 | t_(22)_ = 4.19, p < 0.001 |
| laHC - lPutaman | t_(25)_ = 3.8, p = 0.001 | t_(22)_ = 3.99, p = 0.001 |
| laHC – rpreCG | t_(25)_ = 2.3, p = 0.029 | t_(22)_ = 2.62, p = 0.015 |

**Table S1**. Comparing older and younger adults' RSFC, separately for males and females. All the connections showed lower RSFC for older than younger adults, both for males and for females. Abbreviations: l=left, r=right, aHC=anterior hippocampus, pHC=posterior hippocampus, IPL=inferior parietal lobule, vmPFC=ventromedial prefrontal cortex, PCC= posterior cingulate cortex; preCG=precentral gyrus.

**Group Differences in Head Movements as a Possible Confounder**

Head movements are known to influence fMRI signal. Although participants with large movements were discarded from analysis and head movements were regressed out during RSFC analysis (see Methods section), it is still possible that systematic differences between the groups in head movements caused group differences in the RSFC. To test this possibility, we compared the head movements of older and younger adults during rest. To this end, for each participant we calculated the averaged movements across both rest runs, separately for each movement axis. These measures were entered into a 2-way mix analysis of variance (ANOVA) with age (older/younger) and axis (x, y, z, rotation x, rotation y and rotation z) as independent variables. Results showed that the effect of age was non-significant (F_(1,49)_ = 0.2, p = 0.6), as was the interaction between age and axis (F_(5,245)_ = 1.3, p = 0.2). These results strengthen the assumption that head movements did not contribute to the differences between the groups in RSFC.

**References**

Andrews-Hanna, J.R., Reidler, J.S., Sepulcre, J., Poulin, R., Buckner, R.L., 2010. Functional-anatomic fractionation of the brain’s default network. Neuron 65, 550–62. doi:10.1016/j.neuron.2010.02.005

Andrews-Hanna, J.R., Smallwood, J., Spreng, R.N., 2014. The default network and self-generated thought: component processes, dynamic control, and clinical relevance. Ann. N. Y. Acad. Sci. 1316, 29–52. doi:10.1111/nyas.12360

Benjamini, Y., Hochberg, Y., 1995. CONTROLLING THE FALSE DISCOVERY RATE - A PRACTICAL AND POWERFUL APPROACH TO MULTIPLE TESTING. J. R. Stat. Soc. Ser. B-METHODOLOGICAL 57, 289–300.

Benjamini, Y., Yekutieli, D., 2001. The control of the false discovery rate in multiple testing under dependency. Ann. Stat. 29, 1165–1188.

Grady, C.L., 2012. The cognitive neuroscience of ageing. Nat. Rev. Neurosci. 13, 491–505. doi:10.1038/nrn3256

Hafkemeijer, A., van der Grond, J., Rombouts, S.A.R.B., 2012. Imaging the default mode network in aging and dementia. Biochim. Biophys. Acta 1822, 431–41. doi:10.1016/j.bbadis.2011.07.008

Moscovitch, M., Cabeza, R., Winocur, G., Nadel, L., 2016. Episodic Memory and Beyond: The Hippocampus and Neocortex in Transformation. Annu. Rev. Psychol. 67, 105–34. doi:10.1146/annurev-psych-113011-143733
